# 2218 An application of the payback framework to evaluate the outcomes of pilot projects supported by the Georgia Clinical and Translational Science Alliance from 2007 to 2014

**DOI:** 10.1017/cts.2018.275

**Published:** 2018-11-21

**Authors:** Latrice Rollins, Nicole Llewellyn, Eric Nehl, Astrid Sosa

**Affiliations:** 1 Morehouse School of Medicine; 2 Emory University

## Abstract

OBJECTIVES/SPECIFIC AIMS: We will use a structured evaluation framework, the payback framework, to document the outcomes of 15 case studies of pilot projects supported by Georgia CTSA from 2007 to 2014. METHODS/STUDY POPULATION: We will use a case study approach including bibliometric analyses of publications associated with the selected projects, document review (e.g., investigator curriculum vitae, biannual project reports) and investigator interviews. RESULTS/ANTICIPATED RESULTS: We will document outcomes in 5 “payback categories”: (1) knowledge, (2) research targeting, capacity building, and absorption, (3) policy and product development, (4) health benefits, and (5) broader economic benefits. DISCUSSION/SIGNIFICANCE OF IMPACT: This study will aid in characterizing the returns resulting from this research funding and identify its strengths and weaknesses. This study will inform our understanding of the diversity and breadth of outcomes resulting from Georgia CTSA-supported research, and the value pilot projects provide to clinical and translational science and the broader community.

